# Fast Identification and Removal of Sequence Contamination from Genomic and Metagenomic Datasets

**DOI:** 10.1371/journal.pone.0017288

**Published:** 2011-03-09

**Authors:** Robert Schmieder, Robert Edwards

**Affiliations:** 1 Department of Computer Science, San Diego State University, San Diego, California, United States of America; 2 Computational Science Research Center, San Diego State University, San Diego, California, United States of America; 3 Mathematics and Computer Science Division, Argonne National Laboratory, Argonne, Illinois, United States of America; Universidad Miguel Hernandez, Spain

## Abstract

High-throughput sequencing technologies have strongly impacted microbiology, providing a rapid and cost-effective way of generating draft genomes and exploring microbial diversity. However, sequences obtained from impure nucleic acid preparations may contain DNA from sources other than the sample. Those sequence contaminations are a serious concern to the quality of the data used for downstream analysis, causing misassembly of sequence contigs and erroneous conclusions. Therefore, the removal of sequence contaminants is a necessary and required step for all sequencing projects. We developed DeconSeq, a robust framework for the rapid, automated identification and removal of sequence contamination in longer-read datasets (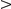
150 bp mean read length). DeconSeq is publicly available as standalone and web-based versions. The results can be exported for subsequent analysis, and the databases used for the web-based version are automatically updated on a regular basis. DeconSeq categorizes possible contamination sequences, eliminates redundant hits with higher similarity to non-contaminant genomes, and provides graphical visualizations of the alignment results and classifications. Using DeconSeq, we conducted an analysis of possible human DNA contamination in 202 previously published microbial and viral metagenomes and found possible contamination in 145 (72%) metagenomes with as high as 64% contaminating sequences. This new framework allows scientists to automatically detect and efficiently remove unwanted sequence contamination from their datasets while eliminating critical limitations of current methods. DeconSeq's web interface is simple and user-friendly. The standalone version allows offline analysis and integration into existing data processing pipelines. DeconSeq's results reveal whether the sequencing experiment has succeeded, whether the correct sample was sequenced, and whether the sample contains any sequence contamination from DNA preparation or host. In addition, the analysis of 202 metagenomes demonstrated significant contamination of the non-human associated metagenomes, suggesting that this method is appropriate for screening all metagenomes. DeconSeq is available at http://deconseq.sourceforge.net/.

## Introduction

High-throughput sequencing technologies have made a huge impact on microbiology, providing a rapid and cost-effective way of generating draft genomes and allowing metagenomic exploration of microbial diversity. Metagenomics, the survey of microbial or viral communities (and their encoded metabolic activities) from distinct environments, has been rapidly expanding over the past several years from its origins in environmental microbiology [1]–[6]. Recently, the National Institute of Health (NIH) roadmap Human Microbiome Project (HMP) initiative was jump-started to examine microbes associated with health and disease in several areas of the human body [7], [8].

Metagenomics has been enabled by the advances in second-generation sequencing, with current sequencing machines generating reads that are shorter than those generated with gel-capillary technology. However, the amount of data produced is orders of magnitude greater than that generated by earlier techniques and can reach gigabases per machine day [9], [10]. The performance characteristics of high-throughput sequencing machines such as Roche/454's GS FLX, Illumina/Solexa's GA IIx, and Life Technologies SOLiD system are changing rapidly with respect to machine capacity, run time, read length, error profile, and cost per base.

The immense amount of genomic and metagenomic data produced today requires an automated approach for data processing and analysis. A typical sequence processing pipeline includes several steps such as sequence cleaning, alignment to known reference sequences, and/or de novo assembly [2], [10]. The sequence cleaning step is an essential first step of the sequence processing pipeline before any further data processing in order to allow accurate downstream analysis. For most datasets, the sequence cleaning step usually includes filtering to remove read duplicates, low quality reads, contaminating sequences, and adaptor or barcode sequences.

Sequences obtained from impure samples or nucleic acid preparations may contain DNA from sources other than the microbes in the sample. That sequence contamination is a serious concern: for the HMP all contaminating human genomic sequences must be removed from the sample prior to the data being made public; for other projects the quality of the data used for downstream analysis will be affected by contamination, possibly causing misassembly of sequence contigs and erroneous conclusions.

In this paper we focus on identifying and removing human contamination from microbial metagenomes, such as those created under the auspices of the HMP. However, the methodology can be applied to any kind of sequence contamination. To detect human contamination, metagenomes need to be compared to the human genome. In addition to the public and private human genome sequencing efforts [11], [12], several individual human genomes were published in the last three years [13]–[18]. Large-scale resequencing projects such as The 1000 Genomes Project (http://www.1000genomes.org/), the Cancer Genome Atlas [19] and the Personal Genome Project (http://www.personalgenomes.org/) will also generate high-coverage human genomes. These projects provide the reference sequences that are used to detect human genome contamination in genomic and metagenomic datasets.

Earlier-generation sequence alignment programs such as BLAST [20], [21] were designed to align DNA and protein sequences and to search through large databases to find homologous sequences. MegaBLAST was developed to speed up the alignment for query sequences that are highly similar to the reference sequences and was used to align large-scale sequencing data. Later, improvements on MegaBLAST were proposed such as database indexing methods to allow even faster alignments [22].

The advances in sequencing technology over the last decade have brought new challenges in bioinformatics; consequently many new alignment programs that are much faster than BLAST have been published over the last few years. In general, the new alignment programs were developed to align DNA sequences to closely related reference genomes, especially long references such as mammalian genomes, with only few low quality alignments expected. For example, many short-read alignment programs were designed for reads 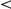
100 bp [23]–[27]. However, most next generation sequencing technologies already produce reads 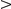
100 bp (Illumina/Solexa), 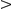
400 bp (Roche/454), and 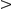
1,000 bp (Pacific Biosciences in early testing [10], [28], [29]). In a few years, long reads will likely dominate and programs for short reads will be less applicable.

In contrast to short-read alignment algorithms that tend to maximize global alignments, longer-read alignment algorithms aim to find local matches because longer reads are more prone to structural variations and map over misassemblies in the reference sequence. Longer-read alignment programs must also be able to deal with alignment gaps since indels (insertions and deletions) occur more frequently in long reads and may be the dominant source of sequencing errors for some technologies such as Roche/454 and Pacific Biosciences [30].

The three approaches used by the currently available longer-read alignment programs are either hash table, suffix tree or Burrows-Wheeler Transform (BWT) [9], [31]. Hash table based algorithms basically follow the seed-and-extend paradigm and the idea of hash table indexing can be traced back to BLAST. The BLAST program keeps the position of each 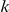-mer subsequence of the query and scans the database sequences for 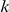-mer exact matches (called seeds) by looking up the hash table. BLAST then extends and joins the seeds and refines them by a Smith-Waterman alignment [32]. The seeding step was accelerated by the idea of requiring multiple seed matches for an extension. This idea is implemented in SSAHA2 [33] and BLAT [34], which offer significantly faster alignment than BLAST for reads that are nearly identical to the reference database.

Recently developed hash table based programs for longer reads such as Mosaik (http://bioinformatics.bc.edu/marthlab/Mosaik) build the hash table on the reference sequences and use it to scan for query subsequences. Hash tables are appropriate for DNA sequences, since they very likely contain repeats or duplicates and are unlikely to contain every possible combination of nucleotides. Depending on the size of the reference sequences, the size of the hash table may be very large (tens of GB) and take a lot of time or memory to build.

Another group of algorithms rely on a representation of suffix/prefix trie. The advantage of using a trie is that an alignment to multiple identical copies of a substring in the reference is only done once since they collapse on a single path in the trie. It takes linear time to determine if a query has an exact match against a trie, but a trie takes quadratic space with respect to the reference length. A suffix tree achieves linear space while still allowing linear-time searching. The alignment program MUMmer [35] is based on suffix tree and anchors the alignment with maximal unique matches (MUMs) and then joins these exact matches with gapped alignments. Most trie implementations require more than 10 bytes per nucleotide and make it impractical to hold the suffix tree of large reference genomes in memory.

The FM-index proposed by Ferragina and Manzini [36] was originally designed as a compressed data structure and is used by alignment programs to improve the memory usage (typically 0.5–2 bytes per nucleotide [31]). The FM-index data structure is basically a compressed suffix array, following the concept that a suffix array is much more efficient if it is created from the BWT sequence rather than from the original sequence. BWT implementations are widely used because of their small memory footprint and they are much faster than their hash-based alternatives at the same sensitivity level [9].

When comparing nucleotide sequences, even a unique query sequence can match a few million positions with a positive alignment score, with the majority being random matches or matches in short low-complexity regions. BWA-SW [30], an implementation of the Burrows-Wheeler aligner combined with a Smith-Waterman search uses heuristics to accelerate the alignment process. BWA-SW traverses the query prefix directed acyclic word graph (DAWG) in the outer loop and the reference prefix trie in the inner loop. From this, all the nodes in the reference prefix trie that match the query node with a positive score are found. Since the true alignment tends to have a high alignment score, it is possible to prune low-scoring matches at each node, and consequently restrict dynamic programming around good matches only. At each node in the DAWG, BWA-SW only keeps the top 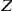 best-scoring nodes in the reference trie that match the node, rather than keeping all the matching nodes. This heuristic is referred to as 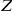-best strategy.

Here, we selected longer-read alignment programs that are actively maintained and widely used and evaluated them on simulated datasets. These programs include BLAST, BLAST+ [37], Mosaik, NUCmer (from MUMmer package), and BWA-SW. Based on the evaluation results, we adopted BWA-SW for the removal of human sequence contamination from metagenomes and developed DeconSeq, a robust framework for the rapid, automated identification and removal of sequence contamination from longer-read datasets. DeconSeq is implemented in Perl and is freely available at http://deconseq.sourceforge.net/. Using DeconSeq, the amount of possible human DNA contamination in 202 metagenomes was investigated.

## Results

### Comparison of program performance

**Figure 1:**
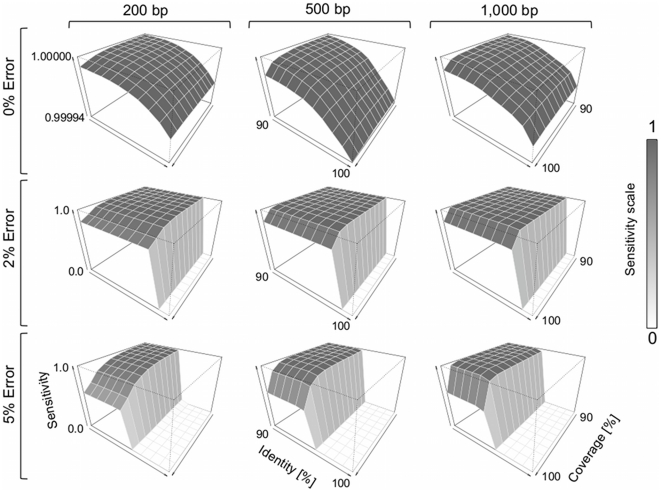
Alignment sensitivity of BWA-SW for human sequences. Query coverage and alignment identity values ranged from 90% to 100%. The sensitivity shows how many sequences could be aligned back to the reference. The simulated datasets contained 28,612,955 reads for 200 bp, 11,444,886 reads for 500 bp, and 5,722,210 reads for 1,000 bp.

The programs Mosaik, NUCmer, BLAST, BLAST+ and BWA-SW were compared for their ability to perform the alignments of the simulated longer-read metagenomes against the human sequence database (see Text S1 for details). Overall, BWA-SW performed with the lowest running time of approximately 22 minutes for the human simulated datasets and four minutes for the bacterial and viral simulated datasets (see Text S1, Figure 1). We did not include BLAT and SSAHA2 in our comparison as these programs were compared to BWA-SW previously and showed similar or worse sensitivity with much longer time spend for the computations [30].

Based on the comparisons, we tentatively adopted BWA-SW to identify and remove human contamination from metagenomes. BWA-SW was the fastest algorithm to complete the identification of 100,000 human sequences using the available computational resources. However, speed and computational requirements are only one aspect of the identification of possible contaminating sequences. The program must also be able to accurately identify all of the contamination in the sample. We therefore assessed the sensitivity of the BWA-SW algorithm at identifying human DNA contamination. This test also identified which sequences the aligner commonly missed.

### Evaluation of alignment sensitivity

There are known limitations to the alignment approach such as placing reads within repetitive regions in a reference genome. BWA-SW was evaluated for its ability to align simulated data containing sequences extracted from the human reference genome back to the reference genome. The simulated data contained 200 bp, 500 bp, or 1,000 bp long sequences. Errors were introduced at rates of 2% and 5%. The typical error rate for real data is approximately 0.5%, therefore this analysis provides a worst-case scenario [38]. The human reference genome was used for this analysis, because it presents the only finished-grade human genome sequence available [10]. The human reference genome was constructed from multiple individuals, contains 2.86 Gbp, covers 99% of the human genome with 357 gaps and has an estimated error rate of 1 in every 100,000 bp (http://www.ncbi.nlm.nih.gov/projects/genome/assembly/grc/human/data/?build=37).

**Figure 2:**
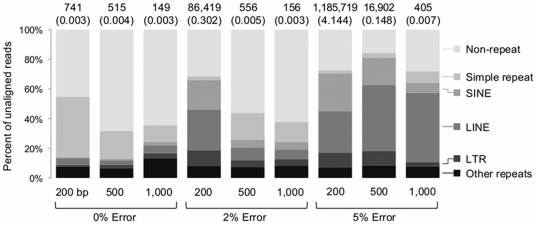
Repeats causing alignment problems for BWA-SW. The query coverage was set to 95%, with identity set to 99%, 97% and 94% for error rates of 0%, 2% and 5%, respectively. The numbers above the bars show the number of unaligned sequences of each category for the given thresholds. The values shown in parenthesis represent the percentage of unaligned sequences. The simulated datasets contained 28,612,955 reads for 200 bp, 11,444,886 reads for 500 bp, and 5,722,210 reads for 1,000 bp.

After computing the alignments, we filtered the results based on query coverage and alignment identity values (Figure 1). Using the default settings, longer sequences could be aligned to the correct region more often than shorter sequences independent of the error rates introduced. Without using alignment thresholds, more than 99.9% of all sequences could be aligned back to the reference. Of the simulated sequences that did not match the reference with the given thresholds, on average more than 56% of the sequences were derived from repeat regions of the human reference genome (Figure 2). Simple repeats and low complexity regions that represent the majority of the unaligned sequences with 0% error rate cover 0.84% and 0.55% of the human reference genome, respectively [39]. In contrast, the unaligned sequences were rarely from regions of the human genome that contained exons (on average less than 4%).

**Figure 3:**
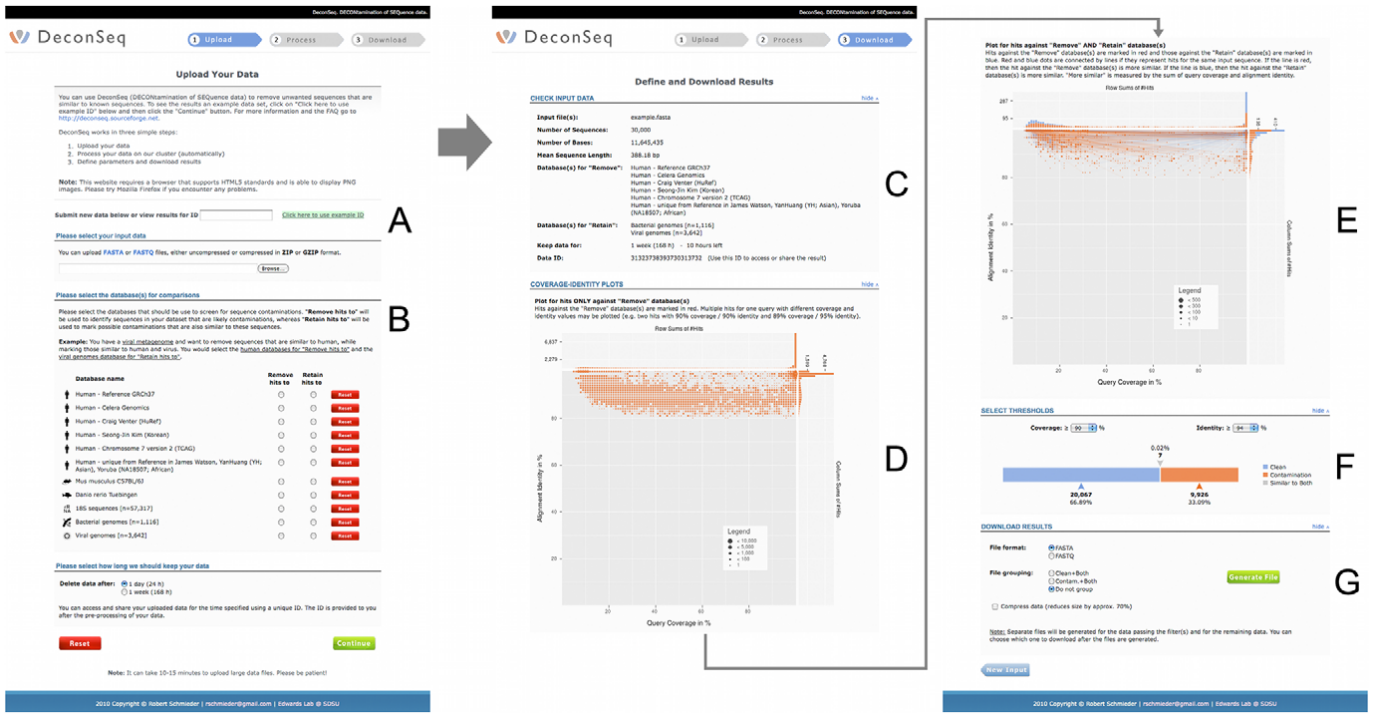
DeconSeq web interface. Screenshots of the DeconSeq web interface at different steps of the data processing. The user can either input a data ID to access already processed data (A) or input a new sequence file and select the database (B). After processing the data, the results are shown including the input information (C), Coverage vs. Identity plots for “remove” databases (D) and “retain” databases (E), classification of input data into “clean”, “contamination”, and “both” (F), and download options (G).

**Figure 4:**
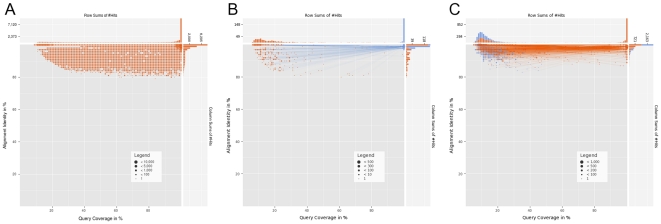
Coverage vs. Identity plots generated by DeconSeq. The plots show the number of matching reads for different query coverage and alignment identity values. The size of each dot in the plots is defined by the number of matching reads with exactly this coverage and identity value. Red dots represent matching reads against the “remove” databases and blue dots against “retain” databases. The column and row sums at the top and right of each plot allow an easier identification of the number of sequences that match for a particular threshold value. The plots for matching reads against the “remove” databases do not show matching reads that additionally have a match against the “retain” databases (A). Results for reads matching against both databases are shown in a second plot where dots for a single read are connected by lines. If the match against the “remove” database is more similar, then the line is colored red, otherwise blue. In B, for example, the majority of sequences is more similar to the “retain” databases and in C the majority is more similar to the “remove” databases.

The sequences that could not be aligned under the given thresholds were then aligned against the same human genome using higher 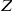-best values (ranging from two to ten) or additional human genome data. Increasing the 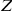-best value increased the number of unaligned sequences that could be aligned (see Text S1, Figure 2). However, using higher 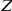-best values almost linearly increases the runtime (see Text S1, Figure 3). Using additional human genome data as reference increased the number of unaligned sequences that could be aligned (see Text S1, Figure 4) using the default 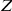-best value.

### Evaluation of DeconSeq accuracy

Alignments are scored based on their query coverage and alignment identity percentages. Only hits above both thresholds are considered as valid alignments in the following evaluation. The accuracy of DeconSeq was benchmarked using simulated metagenomic data because “real” metagenomes lack the correct annotation for all sequences. Metagenomes consist of sequence fragments derived from the available genomes in the sampled environment [40]. To simulate metagenomes, we extracted sequences from completely sequenced genomes and simulated substitution and indel errors (see Methods). DeconSeq was additionally benchmarked using artificial microbial metagenomes obtained from the Joint Genome Institute [41]. The human genomes were used as “remove” databases and the bacterial and viral genomes as “retain” databases.

The accuracy values were calculated for threshold values of 95% query coverage and varying alignment identity. For identity thresholds of 94% and 97%, more than 99.9% of each simulated metagenome were classified correctly (Table 1). For an identity threshold of 99%, the human metagenomes were classified correctly with lower accuracy, caused by the lower number of possible matching sequences due to the introduced error rate above 1% using a 1% average error rate. Variation in read length did affect the accuracy of DeconSeq in identifying contaminating sequences, as mainly short sequences were misclassified.

**Table 1:** Accuracy of DeconSeq for identifying human DNA contamination in simulated metagenomic datasets.

Metagenome group	Accuracy (in %) for identity threshold of		
	94%	97%	99%
Virus	99.9997 ( 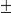 0.0027)	99.9994 ( 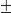 0.0054)	99.9990 ( 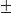 0.0060)
Human	99.9834 ( 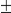 0.0086)	99.9293 ( 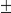 0.0177)	72.3199 ( 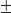 0.2389)
Bacteria	100 ( 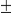 0.0000)	100 ( 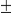 0.0000)	100 ( 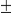 0.0000)
Bacteria JGI	99.9999 ( 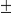 0.0008)	99.9999 ( 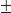 0.0008)	99.9999 ( 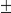 0.0008)

The accuracy values are average values of ten viral, ten microbial and ten human datasets with 100,000 sequences each and three microbial simulated metagenomes from JGI [41]. The accuracy values are shown for threshold values of 95% query coverage and varying alignment identity. The low accuracy value for the human datasets and 99% identity threshold was caused by the lower number of matching sequences due to the introduced errors above 1%.

### Standalone and web application

DeconSeq is publicly available as standalone version or through a user-friendly web interface (Figure 3). The interactive web interface facilitates navigation through the results, definition of threshold parameters, and allows the export of the results for subsequent offline analysis. The input page of DeconSeq provides a mechanism to import new datasets and to select the contamination databases. Users can choose between submitting and processing a new dataset or accessing already processed datasets using a unique identifier. The web interface additionally provides graphical visualizations of the alignment results and the number of reads classified as contamination. The coverage vs. identity plots (Figure 4) can guide the users in their threshold selection. The connected dots in these plots help to identify possible contaminant sequences from non-contaminant sequences that match against both the “remove” and “retain” databases.

### Identification of human contamination in 202 metagenomes

**Figure 5:**
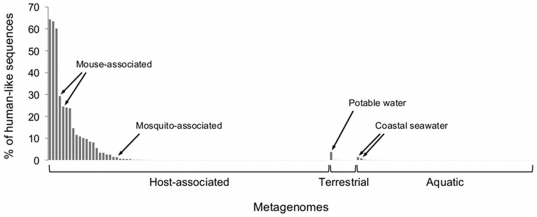
Result of human DNA contamination identified in 202 metagenomes. All seven human genome sequences were used as “remove” databases and depending on the metagenome type (viral or microbial), the viral or bacterial genomes were selected as “retain” database. 145 (72%) of the metagenomes contained at least one possible contamination sequence using a threshold of 95% query coverage and 94% alignment identity.

In an application example, DeconSeq was applied to 202 longer-read metagenomic datasets previously published and with a mean read length greater than 150 bp (see Table S1). Metadata was either retrieved with the data from NCBI or through manual literature search. No prior knowledge of the amount of human contamination was assumed. The FASTA files were provided as input and the human databases were selected as “remove” and bacterial and viral databases were selected as “retain” for microbial and viral metagenomes, respectively. The results of the human contamination identified are summarized in Figure 5. The human contamination was identified for up to 64% of the metagenomes using the thresholds of 95% query coverage and 94% alignment identity. The host-associated metagenomes showed the highest fraction of likely human contamination. Of all metagenomes, 145 (72%) contained at least one possible contamination sequence. The two mouse-associated metagenomes with 24% and 29% possible human contamination were further compared to the mouse reference genome C57BL/6J build 37 to investigate if the high amount of possible contamination is host-related or of human origin. The two metagenomes contained 56% and 57% mouse-like sequences, respectively.

## Discussion

Sequence contamination is a serious concern to the quality of genomic and metagenomic data used for downstream analysis. Therefore, it is important to process sequence data before analyzing it. We presented a program available as either standalone or web-based application that implements features to improve the quality of sequence datasets by identifying and removing possible sequence contamination with high accuracy. The program is targeted towards longer-read datasets and able to process next-generation sequence datasets with gigabases of data.

There are different approaches on how to identify sequence contamination in genomic and metagenomic datasets and the current methods have critical limitations. The dinucleotide relative abundance was used by Willner et al. [42] to predict if a metagenome was contaminated. However, this approach only allows the identification of contamination in the whole dataset, not on the level of single sequences. Others used BLAST to compare metagenomes to the human reference genome [43], [44]. BLAST has a speed disadvantage that makes it a bottleneck for analyzing the huge amounts of data typical of current sequencing projects. The identification of possible contamination based on sequence alignments, however, seems to provide the only reliable option currently available to classify single sequences as contamination.

A major limitation of the alignment approach is the lack of corresponding regions that do not exist in the reference genome(s), referred to as dark matter, which may result from gaps in the reference or the presence of structural variants in the genome(s) being analyzed [45], [46]. Li et al. [47] have found the presence of extensive novel sequences in recently sequenced human genomes that were absent from the human reference genome. Therefore, DeconSeq provides databases for the identification of human DNA contamination from the seven currently available human genomes. More genomes will be sequenced, for example, in large-scale resequencing projects such as The 1000 Genomes Project to enhance our understanding of how genetic differences affect health and disease. This will provide a resource for a more complete human decontamination reference database.

The choice of the alignment program depends on the biological application and on the type of sequencing technology used to generate the data. The scalability of an alignment program for speed and memory usage is important as neither memory nor CPU power is growing as fast as sequencing capacity. The ability to align sequences of different lengths is an important factor as well, considering the rapidly evolving field of next-generation sequencing and the constantly increasing length of produced reads.

As shown in this study, BLAST does not scale well for the identification of human DNA contamination in next-generation sequencing data. Using filters for low complexity or repeat regions may significantly reduce the resources consumed, but also decrease sensitivity. BWT-based alignment programs are more efficient on very long reference sequences such as mammalian genomes because their complexity is better than being linear to the reference length. On bacterial genomes however, these aligners might be slower than hash table based alignment programs. Furthermore, alignment programs such as BWA-SW can be used for sequences generated by methods ranging from pyrosequencing and Sanger sequencing to single molecule sequencing. Such programs allow the alignment of sequence reads of more than 1 Mbp.

The ability to map sequence reads uniquely to the correct location is dependent on a number of factors such as the complexity of the reference data (highly polymorphic or repetitive regions), length of the sequence reads, error rates of the reads, and the diversity of the individual organism compared to the reference [23], [48]. Wrong alignments may be caused by overlooking alignments with a similar score to the best reported alignment.

Different alignment programs handle the issue of reporting unique hits or multiple hits differently. The implemented algorithm either randomly chooses one, reports all above a cutoff, or those with the best alignment score. When errors are introduced, reads might match better at a different locus than the original one, and therefore evaluations of programs with real data containing errors are challenging. The number of suboptimal hits may help to decide which alignments are reliable. In practice however, only the best alignment is used in the analysis [49]. To identify contamination, it is sufficient to find a single match above given thresholds without calculating all possible matches.

Smith et al. [50] found that using base quality scores improves alignment accuracy if the aligner uses lower penalties for an error-prone mismatch. However, accurate quality scores are not always available and the only program evaluated in this study that is able to incorporate quality scores into the alignment algorithm did not fulfill the system requirements. The algorithm implemented in BWA-SW does not make use of quality data, but the quality information could be exploited to estimate the confidence in an alignment.

Not all sequences that should have been aligned might have been aligned using a given program. In some instances, the sequence read cannot be mapped to the reference. Most of these errors arise from failing to find a seed during the mapping step of the algorithm. Repeat regions are problematic for alignment algorithms and users tend to mask sequences before performing the alignments. However, not allowing seeding in matching regions of the reference sequence that are masked for repeats might result in unaligned query sequences. The human reference genome build 37 has 50.2% of the genome masked as repeat, reducing the number of possible seeding positions. It is more likely to find sufficient seeds from which to extend the alignment for longer reads. As read length increases, the mapping in repetitive regions will improve. We showed that the BWA-SW program used by DeconSeq has a high sensitivity (including repetitive regions) for sequences with low error rates or longer reads when aligning human DNA to the reference genome.

Heuristics present another source of alignment errors especially for short queries, because only a few valid unique seeds may exist between the aligned sequences. BWA-SW, for example, tends to miss short alignments with high error rates, as it does not guarantee to find all local hits due to the heuristic acceleration. In contrast, BWA-SW might find seeds where other programs, such as BLAST, do not. BLAST uses identical seeds that might not work well for (short) query sequences that contain mismatches because there might be no seed sequence from which to extend the alignment. BWA-SW finds seeds by dynamic programming between two FM-indices and allows mismatches and gaps in the seeds. To achieve higher sensitivity, regions that do not align with a given program can be identified and aligned using more sensitive (and usually much slower) parameters or alternative programs. The default value for 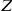 in BWA-SW is one. Increasing 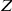 improved accuracy slightly for test datasets, but greatly reduced the alignment speed.

The alignment of sequences against a reference is considered “embarrassingly” parallel, being easy to distribute the required computational work over the nodes of a compute cluster. However, parallelization alone does not always solve the problem of analyzing the huge amounts of data generated by next-generation sequencing machines and speed of the program stays an important factor when choosing programs. We showed that the identification of contaminating sequences done by BLAST+ in hours could be achieved by BWA-SW in minutes. Speed is gained in BWA-SW largely from the use of FM-indices and by reducing unnecessary extension for highly repetitive sequences [30]. However, the speed of alignments is largely determined by the error rate of the query sequences. The error rates will likely be reduced greatly using third-generation sequencing techniques, such as single-molecule techniques that are able to sequence the same template molecule more than once and produce a consensus read with reduced stochastic errors that may occur [10].

It is important that the limitations of the programs used for analysis be understood. Next-generation alignment programs were mainly designed for DNA alignments implementing a 2-bit representation of sequences. The 2-bit representation restricts the use of ambiguous bases such as N. SSAHA2 replaces ambiguous bases by base A and BWA-SW randomly chooses A, C, G or T as replacement. This can lead to false positive hits especially in long stretches of Ns in genomic sequences. To reduce the number of false positive, we removed long stretches of Ns in the genomes and modified BWA-SW to mismatch Ns in the query sequences during the Smith-Waterman alignment. There are also limitations in speed and accuracy of the BWA-SW program. BWA-SW can be used to align 100 bp reads, but it is slower than using BWA. BWA-SW is less accurate than SSAHA2 on 100–200 bp reads for error rates above 2% [30]. Most 454 libraries, however, have an average read length of 300–500 bp. Additionally, this and other studies [30], [31] show that BWA-SW is up to tens of times faster than existing programs. Next-generation alignment programs are under active development and the performance and feature set of each of these programs is likely to improve. If the loss of sequence data can be afforded, reads with high error rates (for example containing low base quality scores or ambiguous bases) and short reads should be filtered prior to using DeconSeq to ensure high accuracy of the contaminant classifications.

The BWA-SW program was modified to fit the needs of DeconSeq. Those modifications do not change the default behavior of the algorithm and are only forced using additional parameters. The default SAM output contains data that is not needed for DeconSeq and usually generates huge output files for reference datasets with a large number of sequences. Furthermore, the Cigar string (a human readable alignment string) presented the only resource in the SAM output from BWA-SW that could be used to calculate coverage and identity values of the alignments. However, the Cigar string uses “M” for matching positions and replacement (mismatch) positions. This would require realigning the sequences in the regions specified by “M” to retrieve the number of replacements used for alignment identity calculations. The mapping quality in the SAM file did not present a sufficient value for the use as threshold. In any case where there are two or more equally likely alignments (multiple locations a query can map to), the mapping quality is zero. This may occur for the repeat-rich human genome and equally likely alignments can still represent contaminating sequences.

DeconSeq uses coverage and identity thresholds to determine if a match is a possible contamination or not. This approach is based on the idea that looking for similar regions consists of grouping sequences that share some minimum sequence similarity over a specified minimum length. It is important that the limitations of this approach be understood. The approach invariably leaves out related regions that have degraded over time, so their similarity is below the threshold. Moreover, the thresholds chosen to group elements together often have no connection to evolutionary history and the underlying mechanisms of formation. For example, the operational definition of segmental duplications excludes ancient duplications that were formed by the same mechanisms long ago but that have since degraded below 90% sequence identity [39]. There is no “one-size-fits-all” solution and each user must make informed decisions as to the appropriate thresholds used for decontamination. Thresholds should not be set to 100% if errors are expected in the sequence reads.

To our knowledge, DeconSeq is the first program optimized to automatically identify and remove sequence contamination from large sequence datasets. In order to avoid the classification of non-contaminating sequences as contamination, all possible contamination can be compared to a second set of databases and marked accordingly. This is especially useful for the identification of human contamination in viral metagenomes because there are a large number of viral or viral-like sequences hidden in the human genome. It is important to note that viral or bacterial sequences of unknown origin but highly similar to the human genome will be classified as contaminants due to the missing reference sequences in the second set of databases.

We evaluated the classification of contamination using simulated datasets and showed that DeconSeq performed with very high accuracy. The highest levels of possible contamination in 202 previously published microbial and viral metagenomes were found in host-associated metagenomes suggesting DNA extraction issues rather than contamination introduced during sample processing.

Next-generation sequencing data is available to most small laboratories. However, they do not always have access to the required computing resources. The web-based version of DeconSeq allows users to conduct decontamination using our in-house computing resources and provides additional visualizations such as coverage vs. identity plots to help users choose the best thresholds for their datasets. Furthermore, the web-based version provides the latest versions of a variety of datasets such as human genome sequence assemblies. Users can contact the authors and request additional databases for specific decontamination purposes.

## Design and Implementation

### Reference data

The human reference genome build 37, the Celera Genomics human genome assembly, the J. Craig Venter genome (HuRef), and The Center for Applied Genomics (TCAG) human chromosome 7 version 2 assembly were downloaded from National Center for Biotechnology Information (NCBI). The Korean male (Seong-Jin Kim; SJK) genome data was retrieved from KOBIC. The Asian male (Han Chinese individual; YH) genome data was retrieved from the YanHuang database. The unique James D. Watson sequences were downlaoded from NCBI and the unique Asian (YH) and unique Yoruban male (NA18507) sequences were downloaded from the supplemental material of Li et al. [47]. All unique sequences were filtered to remove sequence copies and only keep sequences with at least 300 bp. The bacterial genomes (1,116 genomes in 2,103 fasta files as of 06/06/2010) and viral genomes (3,642 genomic sequences as of 06/06/2010) data was retrieved from NCBI. The gene and repeat annotations for the human reference genome build 37 were downloaded from the UCSC Genome Browser [51]. The amount of the genome that was repeat-masked was calculated based on all non-ambiguous bases. A more detailed description including links can be found in Text S1.

### Simulated metagenomes

The program Grinder version 0.1.8 (http://sourceforge.net/projects/biogrinder/) was used to create simulated human, bacterial and viral metagenomic sequences. Sequences were generated using an average error rate of 0.85% substitutions and 0.15% indels (-m 0.85 0.15), and normal distributed read lengths with a mean of 380 bp and standard deviation of 100 bp (−l 380 normal 100). Simulated sequences were then filtered using PRINSEQ [52] to generate ten human, ten bacterial and ten viral datasets with 100,000 unique sequences containing no Ns and a read length of at least 100 bp.

Additionally, three artificial microbial metagenomes with different complexity obtained from the Joint Genome Institute (JGI; http://fames.jgi-psf.org/) were used [41]. The JGI metagenomes were pre-processed using PRINSEQ to trim poly A/T tails longer than 10 bp and to remove reads shorter than 100 bp and exact sequence duplicates. The resulting three datasets contained 116,739, 97,479 and 114,430 sequences with a mean read length of 948.5 bp, 950.9 bp and 966.8 bp, respectively.

### Human reference datasets

The Human reference genome build 37 was used to analyze the type and amount of unaligned sequences using BWA-SW. Datasets with sequences of 200 bp, 500 bp and 1,000 bp length were generated from the reference genome sequence using 50% overlap. All sequences that contained the ambiguous base N were discarded as N aligned to N is considered a mismatch and would alter the alignment identity for identical sequences. The resulting datasets contained 28,612,955 reads for 200 bp, 11,444,886 reads for 500 bp, and 5,722,210 reads for 1,000 bp. Error rates of exactly 2% and 5% (with 15% indels and 85% substitutions) were then simulated for each of the three datasets resulting in 6 additional datasets.

### Reference databases for web-based version

The web-based version offers pre-processed reference databases for a variety of complete genomes such as human, bacterial and viral genomes. The genome data was preprocessed before indexing using BWA. To reduce the number of false positive matches that might be introduced due to the long stretches of Ns that will be randomly replaced by A, C, G or T during database indexing, the genome sequences were split at stretches of 200 or more Ns. The separated sequences were then filtered for read duplicates to reduce redundancy in the sequence data and for short sequences that contained more than 5% of ambiguous bases (N). In its current version, BWA-SW fails to index the complete dataset from multiple human genomes (BWTIncConstructFromPacked error). However, the error was not a concern for the web-based version because to decrease the memory usage on the computing cluster, the genome data was split into smaller files that require a maximum of 1.5 GB of memory per chunk. The results for the split databases are automatically joined before generating the output for the web-based version of DeconSeq.

### Implementation and computational platform

**Figure 6:**
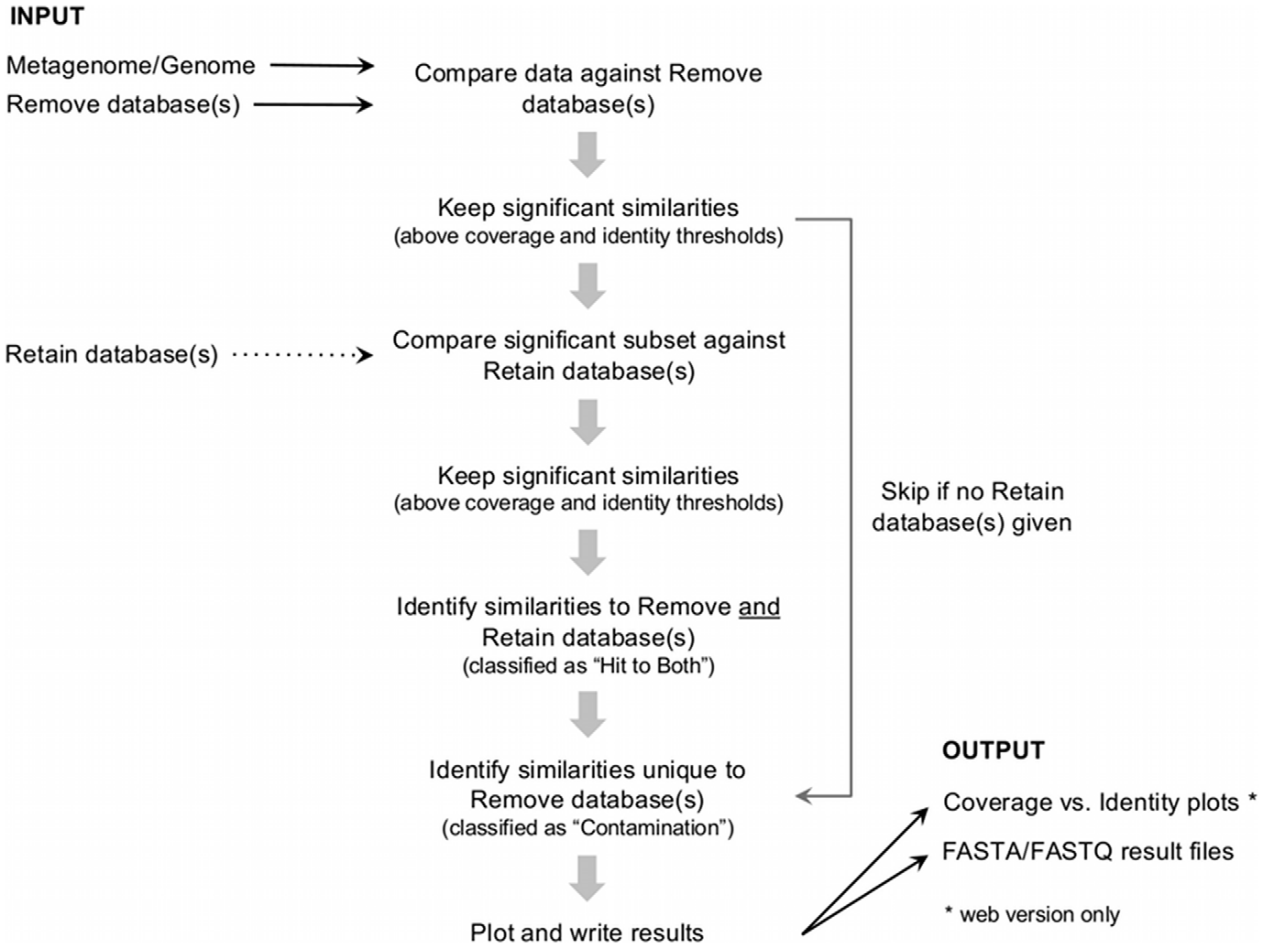
Flowchart of DeconSeq for the identification of possible contaminant sequences.

DeconSeq was implemented as standalone and web-based version in Perl. The workflow of DeconSeq is shown in Figure 6. The DeconSeq web application is currently running on a web server with Ubuntu Linux using an Apache HTTP server to support the web services. The web interface provides a high level of compatibility with heterogeneous computing environments. The alignments are computed on a connected computing cluster with ten working nodes (each with 8 CPUs and 16 GB RAM) running the Oracle Grid Engine version 6.2. The input data is automatically split into chunks for optimized distribution of work over the working nodes.

### Modifications of BWA-SW

The BWA-SW source code was modified to fit the requirements for DeconSeq. The file bwtsw2_aux.c was modified to generate an alternative output, which presents a lightweight tab-separated output format containing only the necessary data required by DeconSeq (query identifier, reference identifier, query coverage and alignment identity). The file bwtsw2_aux.c was additionally modified to force a mismatch when aligning the ambiguous base N in query sequences instead of randomly replacing it by A, C, G or T and possibly resulting in a match (BWA-SW default). The files stdaln.c, stdaln.h and bwtsw2_aux.c were modified to include “R” for replacements in an extended version of the Cigar string, instead of using “M” for both match and replacement (mismatch). The files bwtsw2_main.c and bwtsw2.h were modified to fix the double defined parameter -s (changed to -s and -S), and to add the new parameters -A (generate alternative output), -R (output extended version of Cigar string with replacements) and -M (force to mismatch Ns in query sequence). The modified version of BWA-SW is made available as part of the DeconSeq source.

### Input and output

The input for DeconSeq is FASTA formatted data containing the genomic or metagenomic reads. In addition to FASTA files, the user can submit FASTQ files (containing sequence and quality data) [53] using the web interface. The BWA-SW algorithm does not make use of quality data during the alignment of sequences and does therefore not require quality data as input. The input data is checked to be a valid file with DNA data. If the input data fails the validation step, further processing is restricted.

Sequence files can be of large size (several 100 MB), and therefore the web interface additionally allows the submission of compressed FASTA or FASTQ files to reduce the time of data upload (by approximately 70%) from the user machine to the web server. Currently, ZIP and GZIP compression algorithms are supported. If the compressed files contain more than one FASTA or FASTQ file, the single files will be joined into one dataset. The file formats and compression types are automatically detected and processed accordingly. There is no limit on the number of sequences or the size of the input file accepted by DeconSeq.

The web-based version of DeconSeq offers several pre-processed databases to select from for the two categories of “remove” and “retain”. Databases are available, for example, for the seven publicly available complete human genomes, as well as for groups of bacterial and viral genomes. The databases used for the web-based version are automatically updated on a regular basis. The user can download the results in the web interface in FASTA or FASTQ (if provided as input) format or its compressed version. The results can either be separated or joined files. This allows the user to further investigate the results separately. Results will be stored for the time selected by the user (either one day or one week), if not otherwise requested, on the web server using a unique identifier displayed during data processing and on the result page. This identifier allows the user to share the result with other researchers without having to re-submit and re-process the dataset.

### Filter and threshold parameters

The user can filter the data based on different parameters. Unlike the standalone version, the web-based program allows the user to define filter parameters based on the input data after the data is processed. This does not require an *a priori* knowledge of the best parameters for a given dataset and the parameter choice can be guided by the graphical visualization of the results.

Sequences are classified as contamination if they have a match above the threshold values against any database selected for “remove”. The thresholds are based on query coverage and alignment identity to allow an unbiased filtering for diverse datasets and databases, in contrast to using unnormalized E-values or alignment scores. Threshold values are rounded toward the lower integer (e.g. 99.95% is rounded to 99%). In order to avoid the classification of non-contaminating sequences as contamination, all possible contaminating sequences can be compared to alternative databases (“retain” databases) and matches above the thresholds are marked accordingly with “Hit to both”.

### Analysis of 202 metagenomes

The amount of possible human DNA contamination in 202 longer-read metagenomes (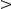
150 bp mean read length) was estimated using DeconSeq. These metagenomes were previously published and are publicly available from NCBI (http://www.ncbi.nlm.nih.gov/). The metagenomes used here represent viral and bacterial communities sampled from a diverse array of biomes and were categorized as one of the following: “aquatic”, “terrestrial”, and “host-associated”. The metagenomes were further subdivided into their sampled environment, such as “human”, “mouse”, and “soil”. Sampling, filtering, processing and sequencing methods differed among the compiled metagenomes. Table 2 provides a summary of the number of metagenomes from each type and biome (a more detailed list of the complete dataset can be found in Table S1).

**Table 2:** Summary of metagenomes by type and biome used in this study.

Biome	Number of viral metagenomes	Number of microbial metagenomes
Aquatic	1	58
Terrestrial	9	6
Host-associated (total)	65	63
Host-associated (human)	62	50
**Total**	**75**	**127**

The metagenomes were previously published and available through NCBI. The metagenomes were not targeted to a single loci and the mean read length was above 150 bp after trimming and filtering.

The metagenomes used in this study were pre-processed prior to any processing with DeconSeq. UniVec build 5.2 (http://www.ncbi.nlm.nih.gov/VecScreen/UniVec.html) and cross_match (http://www.phrap.org/) were used to screen for vector contamination in the metagenomes. TagCleaner [54] was used to trim adapter and tag sequences. PRINSEQ [52] was then used to filter exact sequence duplicates, sequences shorter than 50 bp or longer than 10,000 bp, sequences containing more than 5% of ambiguous base N after trimming Ns from the sequence ends, and sequences containing non IUPAC conform characters for DNA sequences. The resulting datasets were excluded from the study if the mean sequence length was below 150 bp or the dataset contained less than 1,000 metagenomic sequences. Metagenomes targeted to single loci such as 16S rRNA studies were excluded as well.

For all metagenomes, DeconSeq was run using all human databases for “remove” and depending on the type (microbial or viral) the bacterial or viral genomes database was selected for “retain”. The threshold values were set to 95% coverage and 94% identity.

### Calculation of sensitivity and accuracy

Sensitivity (or true positive rate) was used to evaluate alignment performance for BWA-SW and was calculated for query coverage and identity thresholds ranging from 90% to 100%. Accuracy was used as measurement for the proportion of true classifications by DeconSeq and calculated for thresholds of 95% query coverage and varying alignment identity.
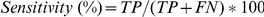
Here, the reads that could be aligned back to the reference sequence were considered true positives (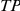). Reads that could not be aligned were considered false negatives (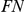).
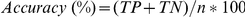
Here, reads that were human and that were classified as human were considered 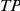. Reads that were non-human and were not classified as human were considered true negatives (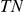). Reads that were classified as “Hit to both” were considered 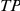 for human reads and 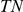 for non-human reads. The number of reads 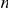 equals to the sum of true positives, false positives, true negatives and false negatives.

## Availability and Future Directions

The DeconSeq standalone version, test datasets, the documentation and the link to the web-based version are available at http://deconseq.sourceforge.net/. All further developments will be made available through this website. Future work will include interface improvements of the web-based version (additional visualizations and filter options) and non-redundant databases to account for the increasing amount of reference genomes containing only a small fraction of new sequence data.

## Supporting Information

Text S1 **Additional methods and results.** This text includes a detailed guide to the retrieval of the reference data and benchmarked programs, the generation of benchmark data, the comparison of program performance for Mosaik, NUCmer, BLAST, BLAST+ and BWA-SW, as well as additional Figures 1, 2, 3, 4.
(PDF)

Table S1 **Details of the 202 metagenomes used for the identification of possible human contamination by DeconSeq.**
(PDF)
