# Supplementary material for: Fast Identification and Removal of Sequence Contamination from Genomic and Metagenomic Datasets
Source: PLoS One. 2011 Mar 9;6(3):e17288. doi: 10.1371/journal.pone.0017288 (PMC3052304; doi:10.1371/journal.pone.0017288)
Supplement: Text S1 — Additional methods and results. This text includes a detailed guide to the retrieval of the reference data and benchmarked programs, the generation of benchmark data, the comparison of program performance for Mosaik, NUCmer, BLAST, BLAST+ and BWA-SW, as well as additional Figures 1, 2, 3, 4. (PDF) [file pone.0017288.s001.pdf]

Reference data

The human reference genome build 37, the Celera Genomics human genome assembly, the J. Craig Venter genome (HuRef), and The Center for Applied Genomics (TCAG) human chromosome 7 version 2 assembly were downloaded from National Center for Biotechnology Information (NCBI) FTP server:

`ftp://ftp.ncbi.nih.gov/genomes/H\_sapiens/Assembled\_chromosomes/`

The data can alternatively be retrieved from the NCBI Genome Project (Project ID: 9558).

The Korean male (Seong-Jin Kim; SJK) genome data was retrieved from KOBIC:

`ftp://ftp.kobic.kr/pub/KOBIC-KoreanGenome/`

The Asian male (Han Chinese individual; YH) genome data was retrieved from the YanHuang database:

`ftp://public.genomics.org.cn/BGI/yanhuang/fa/`

The James D. Watson genome data was retrieved from the NCBI Genome Project (Project ID: 28335; GenBank: ABKV00000000.1):

`http://www.ncbi.nlm.nih.gov/`

This project includes the sequences that are not present in the human reference genome assembled into an accessioned WGS project (ABKV01000000).

The bacterial genomes data was retrieved from the NCBI FTP server with 1,116 genomes in 2,103 fasta files (as of 06/06/2010):

`ftp://ftp.ncbi.nih.gov/genomes/Bacteria/all.fna.tar.gz`

The viral genomes data was retrieved from the NCBI FTP server with 3,642 genomic sequences (as of 06/06/2010):

`ftp://ftp.ncbi.nih.gov/refseq/release/viral/`

`ftp://ftp.ncbi.nlm.nih.gov/genomes/Viruses/all.fna.tar.gz`

The gene and repeat annotations for the human reference genome build 37 were downloaded from the UCSC Genome Browser:

Table browser; genome: human; assembly: GRCh37 assembly; track: RefSeq Genes or RepeatMasker

The repeat-masked human reference genome build 37 was downloaded from the NCBI FTP server:

ftp://ftp.ncbi.nih.gov/genomes/H_sapiens/Assembled_chromosomes/

Non-repeating sequence was formatted in upper case, whereas all repeats were formatted in lower case (lower-case soft-masking). The amount of the genome that was repeat-masked was calculated based on all non-ambiguous bases.

Benchmarked programs

BLAST version 2.2.23 was downloaded from:

ftp://ftp.ncbi.nih.gov/blast/executables/release/LATEST/

BLAST+ version 2.2.23 was downloaded from:

ftp://ftp.ncbi.nih.gov/blast/executables/blast+/2.2.23/

BWA-SW version 0.5.8 was downloaded from:

http://sourceforge.net/projects/bio-bwa/files/

Mosaik version 1.0.1388 was downloaded from:

http://code.google.com/p/mosaik-aligner/

NUCmer (MUMmer) version 3.22 was downloaded from:

http://sourceforge.net/projects/mummer/files/.

Unless necessary, we used the default command-line options of each aligner. Fine-tuning the options based on the characteristics of the input data may yield better performance.

Human reference database for program benchmarks

The reference database used in the program benchmarks with the simulated query metagenomes was build using the human reference genome build 37 and the filtered sequences from the Watson, Asian and Yoruban genome that were not present in the human reference genome. The databases for the different programs were generated using the default parameters of the respective programs. The commands used to generate the human reference database for each of the compared programs can be found below.

Commands used to generate human reference databases

The file human_db.fa contains the sequences of the human reference genome build 37 and the filtered sequences from the Watson, Asian and Yoruban genome that were not present in the human reference genome.

BLAST:

```
formatdb -i human_db.fa -p F -o T -n humanDB -t "Human DB"
-v 4100
```

BLAST+:

```
makeblastdb -in human_db.fa -dbtype nucl -parse_seqids -
hash_index -out humanDB -title "Human DB" -max_file_sz 1GB
```

BWA-SW:

```
bwa index -p humanDB -a bwtsw human_db.fa
```

Mosaik:

```
export MOSAIK_TMP=/tmp/

MosaikBuild -fr human_db.fa -oa humanDB

MosaikJump -ia humanDB -out humanDB_15 -hs 15
```

NUCmer:

No DB generation required by default. To prepare database independent from nucmer:

```
prenuc human_db.fa > humanDB
```

(prenuc can be found in the "aux_bin" directory of mummer)

Comparison of program performance

We required that the programs were able to run on the available resources of 16 GB of memory and 50 GB of hard disk space, as this setup will be used later for production use of the programs. First, the ten simulated datasets with 100,000 human sequences were used as input. This represents our worst-case scenario for a 10\% contamination rate in a dataset of 1,000,000 reads. If the program was able to finish processing the data in a given time frame of 24 hours, it was further evaluated using the bacterial and viral simulated datasets.

Mosaik is a short and long read aligner that accelerates the gapped alignment using a banded Smith-Waterman algorithm. It was the only program used in the comparison that is able to incorporate read quality data for increased alignment accuracy. Mosaik is multi-threaded and well documented. A database for the human reference genome in standard mode required more than 57 GB of memory when performing the alignments and more than 19 GB using a jump database. This alone excluded the program for our purposes. However, we considered the option to separate the database in multiple chunks to fit into the memory of 16 GB (per node) of our system. Unfortunately, we repeatedly run into a problem (segmentation fault error) that was reported and may be fixed in a later release.

NUCmer is part of the MUMmer package and basically presents a wrapper around several MUMmer tools written in Perl. In contrast to all other programs tested, NUCmer does not require an indexed database as input. Instead, NUCmer produces the index during run time. For repeated use of the same sequence data as reference or query, it is possible to modify the NUCmer Perl script to generate the index once, turn off the removal of the index at the end of the script and reuse the same index for the next run. This reduces the pre-processing time of the datasets every time the program is run.

Due to the length restrictions of the input sequences used to generate the suffix tree, we were not able to use the complete human reference genome as input (mummer error: suffix tree construction failed: textlen=2913118792 larger than maximal textlen=536870908). We investigated the alternative of using the reference genome as query instead, as suggested in the program documentation. If a large sequence dataset is required as reference and cannot be used as query, an alternative implementation using maximal exact matches (MEMs [1]) could be used as replacement in NUCmer to remove the length restriction. However, we performed the alignments using the simulated data as reference and the human reference genome as query, and a seed length of 15 (-l 15). Using the simulated data from the human dataset as input, NUCmer finished after an average running time of 4.5 days (109 hours) and was therefore over the time limit of 24 hours. The required memory was 4 GB or less. We experienced that the module ''postnuc'' of NUCmer

required the majority of the run time, probably caused by the many possible hits in the repeat-rich human genome. The modules ''mummer'' and ''mgaps'' were executed for a time of slightly less than 2 hours (out of the 109 hours). To reduce the number of possible matches in repeat regions, a hard-masked genome might be used as input. The repeats should be masked with characters other than N as this will result in matches against the long stretches of Ns in the gap regions of the human reference genome.

BLAST is an earlier-generation sequence alignment program that was designed to align DNA and protein sequences and to search through large databases to find homologous sequences. BLAST has been used for the last 20 years to perform similarity searches and is therefore considered the ''gold standard'' of similarity searches for many researchers. BLAST allows splitting up the database into several files (default 1 GB) and therefore has a small memory footprint. The tabular output of BLAST (parameter -m 8) was used as it provides a space efficient alternative to the standard output. The program was not able to finish the alignments for the human input sequences in the 24 hours time limit. The tabular output did not support the restriction for a maximum number of matching hits to output (parameters -v and -b) and generated GBs of data, possibly slowing down the data processing. To reduce the number of possible matches, the query sequences can be lower case masked in repeat regions. However, this did not sufficiently improve the performance. BLAST does not support soft-masking of repeat regions in the database sequences and the hard-masking of repeat regions with Ns will likely improve performance, but at the cost of much lower sensitivity. We then performed the same search using the MegaBLAST option (-n T). MegaBLAST resulted in errors (Segmentation fault after 4-5 hours) for the human simulated datasets, however it was able to finish the alignment of the bacterial and viral datasets within the time limit.

BLAST+ is a reimplementation of the BLAST program with several improvements and changes. Long query sequences will be broken into chunks and for long database sequences, it is possible to retrieve only the relevant parts of the sequence, reducing CPU time and memory usage for searches of short queries against databases of contigs or chromosomes. BLAST+ additionally allows masking of the database sequences and user defined tabular outputs. The seed length was set to 15 (-word_size 15) for comparable results. BLAST+ required too much memory when using the non-masked database, resulting in lower CPU usage caused by data swapping. The database was therefore re-generated using soft-masked sequence data. The masked database improved the performance and allowed the alignment of the human query dataset with a maximum memory usage of 1 GB. However, the masked database decreased the sensitivity due to unaligned seeds.

BWA-SW is an extension of the BWA program that is based on the BWT and is designed for longer reads with more errors. It performs Smith-Waterman alignments with accelerating heuristics to find high-scoring local hits. BWA-SW is able to identify chimeric sequences. The program automatically adjusts parameters based on the read lengths and sequencing error rate, which is convenient to users not familiar with the algorithm and also helps the performance when the input contains reads of mixed lengths and error rates. With the default settings, BWA-SW returns the best hit only and outputs the results in the SAM format [2]. The program generates an FM-indexed reference database that requires less space than the raw FASTA data and less than 3.4 GB of memory for the human reference genome during the alignment computation. BWA-SW performed with the lowest running time of approximately 22 minutes for the human simulated datasets and four minutes for the bacterial and viral simulated datasets

References

1. Khan Z, Bloom JS, Kruglyak L, Singh M: **A practical algorithm for finding maximal exact matches in large sequence datasets using sparse suffix arrays**. *Bioinformatics* 2009, **25**:1609 -1616.

2. Li H, Handsaker B, Wysoker A, Fennell T, Ruan J, Homer N, Marth G, Abecasis G, Durbin R: **The Sequence Alignment/Map format and SAMtools**. *Bioinformatics* 2009, **25**:2078-2079.

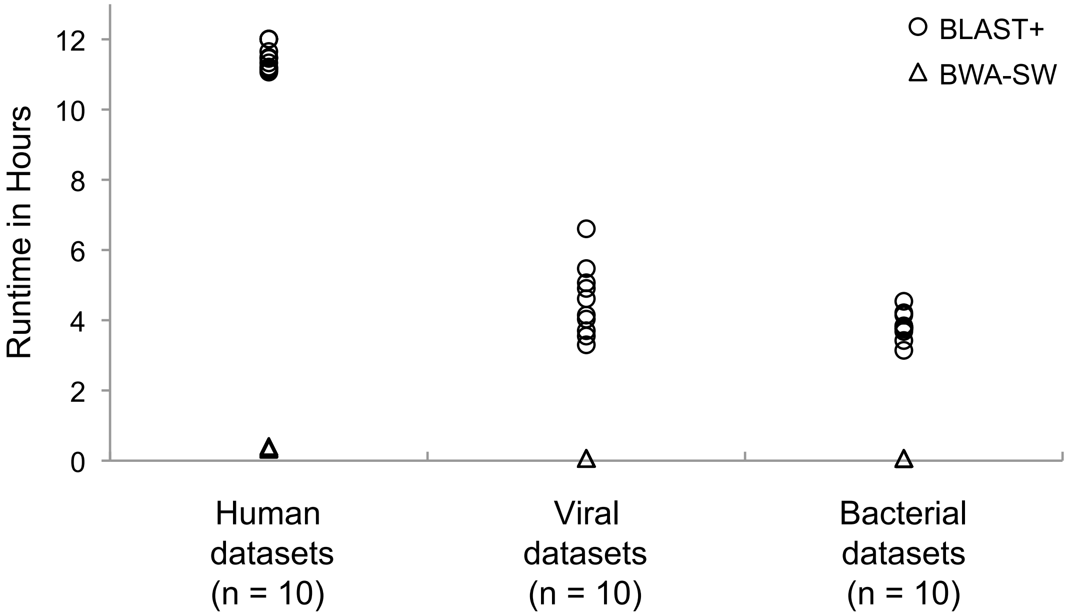

Figure 1: Comparison of runtimes between BLAST+ and BWA-SW

Comparison of the runtimes between BLAST+ and BWA-SW for ten human, viral and bacterial simulated metagenomic datasets. BWA-SW performed with the lowest running time of approximately 22 minutes for the human simulated datasets and four minutes for the bacterial and viral simulated datasets.

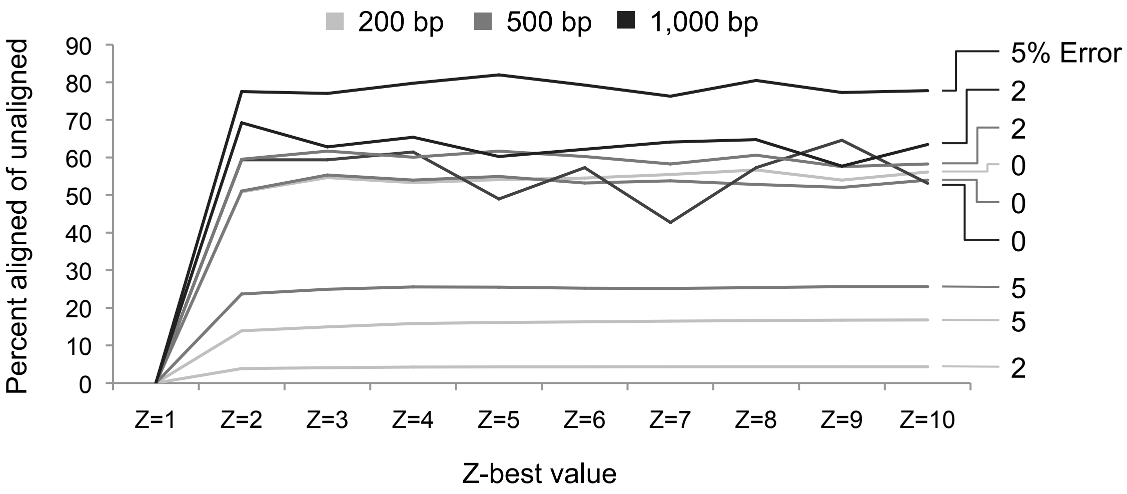

Figure 2: Percentage of unaligned sequences that could be aligned using higher *Z*-best values

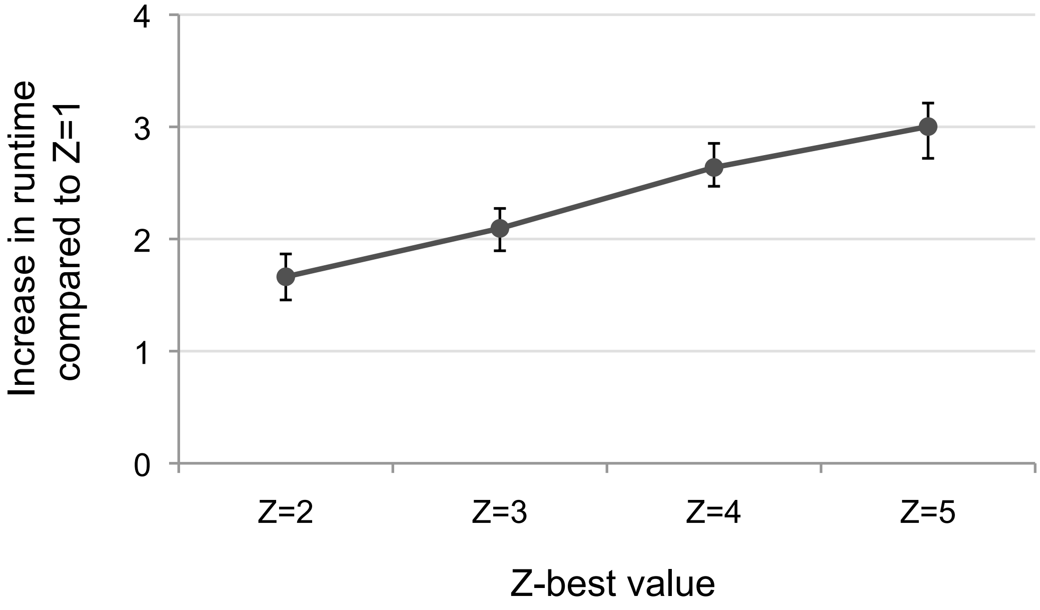

Figure 3: Runtime of BWA-SW for different *Z*-best values

The change in runtime was measured for *Z*-best values ranging from one to five using the 30 simulated metagenomic datasets with 100,000 sequences each. The sequences were compared to the human reference genome and the change in runtime compared to the $Z = 1$ runtime is shown.

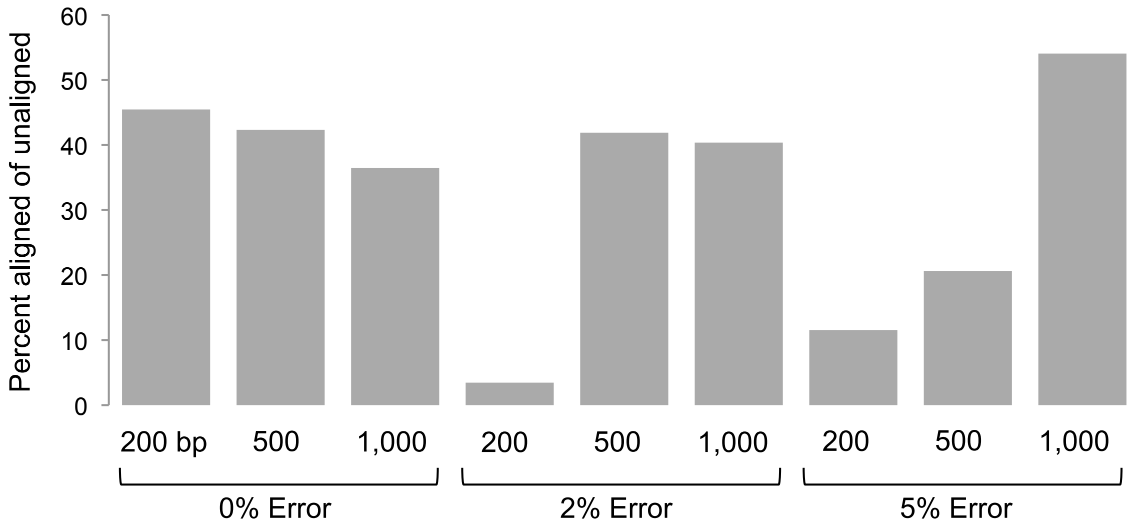

Figure 4: Percentage of unaligned sequences that could be aligned using additional human genome data
