# Supplementary material for: Fast Identification and Removal of Sequence Contamination from Genomic and Metagenomic Datasets
Source: PLoS One. 2011 Mar 9;6(3):e17288. doi: 10.1371/journal.pone.0017288 (PMC3052304; doi:10.1371/journal.pone.0017288)
Supplement: Table S1 — Details of the 202 metagenomes used for the identification of possible human contamination by DeconSeq. (PDF) [file pone.0017288.s002.pdf]

MG_ID	PubMed ID	Type (V/M/E)	Sequencing method	Sample name in publication	Biome	Environment	Sub-classification	# Reads (Filtered)	Mean Length (Filtered)	# Contamination C95-I94	% Contamination
1	16336043	Viral	ABI3730 (Sanger)	Lib 1	Host-associated	Human	fecal	2311	215.41	1	0.04
2	16336043	Viral	ABI3730 (Sanger)	Lib 2	Host-associated	Human	fecal	4358	197.07	64	1.47
3	16336043	Viral	ABI3730 (Sanger)	Lib 3	Host-associated	Human	fecal	3122	198.72	14	0.45
4	16741115	Microbial	ABI3730 (Sanger)	Subject 7	Host-associated	Human	fecal	71581	827.46	13	0.02
5	16741115	Microbial	ABI3730 (Sanger)	Subject 8	Host-associated	Human	fecal	81219	843.85	10	0.01
6	17620602	Viral	GS20 Assembled	TM7a assembly	Host-associated	Human	oral	8543	328.84	58	0.68
7	20333224	Microbial	ABI3730 (Sanger)	M-1m	Host-associated	Human	fecal	1981	665.11	0	0.00
8	20333224	Microbial	ABI3730 (Sanger)	M-11m	Host-associated	Human	fecal	2056	681.17	0	0.00
9	20333224	Microbial	ABI3730 (Sanger)	I-1m	Host-associated	Human	fecal	1778	581.33	2	0.11
10	20333224	Microbial	ABI3730 (Sanger)	I-11m	Host-associated	Human	fecal	2092	679.93	0	0.00
11	17916580	Microbial	ABI3730 (Sanger)	F1-S	Host-associated	Human	fecal	28780	1294.96	0	0.00
12	17916580	Microbial	ABI3730 (Sanger)	F1-T	Host-associated	Human	fecal	36211	1180.99	4	0.01
13	17916580	Microbial	ABI3730 (Sanger)	F1-U	Host-associated	Human	fecal	16156	1277.71	0	0.00
14	17916580	Microbial	ABI3730 (Sanger)	F2-V	Host-associated	Human	fecal	36364	1257.55	0	0.00
15	17916580	Microbial	ABI3730 (Sanger)	F2-W	Host-associated	Human	fecal	29991	1299.66	0	0.00
16	17916580	Microbial	ABI3730 (Sanger)	F2-X	Host-associated	Human	fecal	30927	1200.45	5	0.02
17	17916580	Microbial	ABI3730 (Sanger)	F2-Y	Host-associated	Human	fecal	35059	1290.49	3	0.01
18	17916580	Microbial	ABI3730 (Sanger)	In-A	Host-associated	Human	fecal	19446	1340.48	6	0.03
19	17916580	Microbial	ABI3730 (Sanger)	In-B	Host-associated	Human	fecal	9731	1227.09	0	0.00
20	17916580	Microbial	ABI3730 (Sanger)	In-D	Host-associated	Human	fecal	36763	1211.51	2	0.01
21	17916580	Microbial	ABI3730 (Sanger)	In-E	Host-associated	Human	fecal	19988	1253.84	0	0.00
22	17916580	Microbial	ABI3730 (Sanger)	In-M	Host-associated	Human	fecal	15075	1461.72	20	0.13
23	17916580	Microbial	ABI3730 (Sanger)	In-R	Host-associated	Human	fecal	34693	1247.41	17	0.05
24	19043404	Microbial	GS FLX	F1T1Le1	Host-associated	Human	fecal	217129	238.09	13	0.01
25	19043404	Microbial	GS FLX	F1T2Le1	Host-associated	Human	fecal	440326	178.68	0	0.00
26	19043404	Microbial	GS FLX	F1MOv1	Host-associated	Human	fecal	508663	201.72	5	0.00
27	19043404	Microbial	GS FLX	F2T1Le1	Host-associated	Human	fecal	414067	229.36	18	0.00
28	19043404	Microbial	GS FLX	F2T2Le1	Host-associated	Human	fecal	487258	206.13	77	0.02
29	19043404	Microbial	GS FLX	F2MOb1	Host-associated	Human	fecal	534474	221.05	27	0.01
30	19043404	Microbial	GS FLX	F3T1Le1	Host-associated	Human	fecal	555519	242.79	5	0.00
31	19043404	Microbial	GS FLX	F3T2Le1	Host-associated	Human	fecal	414225	242.64	13	0.00
32	19043404	Microbial	GS FLX	F3MOv1	Host-associated	Human	fecal	499318	249.86	12	0.00
33	19043404	Microbial	GS FLX	F7T1Ob1	Host-associated	Human	fecal	495058	165.52	5	0.00
34	19043404	Microbial	GS FLX	F7T2Ob1	Host-associated	Human	fecal	491044	199.31	13	0.00
35	19043404	Microbial	GS FLX	F7MOb1	Host-associated	Human	fecal	412637	215.04	3	0.00
36	19043404	Microbial	GS FLX Titanium	F10T1Ob1	Host-associated	Human	fecal	301864	335.89	2	0.00
37	19043404	Microbial	GS FLX Titanium	F10T2Ob1	Host-associated	Human	fecal	501314	345.76	6	0.00
38	19043404	Microbial	GS FLX	F10MOv1	Host-associated	Human	fecal	492709	191.31	8	0.00
39	19043404	Microbial	GS FLX	F15T1Ob1	Host-associated	Human	fecal	512517	178.89	34	0.01
40	19043404	Microbial	GS FLX	F15T2Ob1	Host-associated	Human	fecal	545842	204.86	30	0.01
41	19043404	Microbial	GS FLX	F15MOb1	Host-associated	Human	fecal	430549	188.51	21	0.00
42	19156205	Viral	GS FLX	F1	Host-associated	Human	nasal	24839	155.83	14950	60.19
43	19156205	Viral	GS FLX	F2	Host-associated	Human	nasal	20345	153.62	12908	63.45
44	19156205	Viral	GS FLX	F3	Host-associated	Human	nasal	18289	165.04	11772	64.37
45	19156205	Viral	GS FLX	N1	Host-associated	Human	fecal	10495	166.69	4	0.04
46	19156205	Viral	GS FLX	N2	Host-associated	Human	fecal	24357	175.08	5	0.02
47	19156205	Viral	GS FLX	N3	Host-associated	Human	fecal	17884	170.01	2	0.01
48	19156205	Viral	GS FLX	N4	Host-associated	Human	fecal	13441	170.54	1088	8.09
49	19156205	Viral	GS FLX	N5	Host-associated	Human	fecal	22089	176.89	34	0.15
50	19816605	Viral	GS FLX	NonCF1Asthma	Host-associated	Human	sputum	266799	224.1	30753	11.53
51	19816605	Viral	GS FLX	NonCF2	Host-associated	Human	sputum	251533	217.09	59714	23.74
52	19816605	Viral	GS FLX	NonCF3	Host-associated	Human	sputum	226902	229.24	12585	5.55
53	19816605	Viral	GS FLX	NonCF4Spouse	Host-associated	Human	sputum	310921	223.83	74722	24.03
54	19816605	Viral	GS FLX	NonCF5	Host-associated	Human	sputum	311908	213.49	30192	9.68

MG_ID	PubMed ID	Type (V/M/E)	Sequencing method	Sample name in publication	Biome	Environment	Sub-classification	# Reads (Filtered)	Mean Length (Filtered)	# Contamination C95-I94	% Contamination
55	19816605	Viral	GS FLX	CF1	Host-associated	Human	sputum	162552	220.82	16463	10.13
56	19816605	Viral	GS FLX	CF2	Host-associated	Human	sputum	193398	222.28	6519	3.37
57	19816605	Viral	GS FLX	CF3	Host-associated	Human	sputum	162534	238.19	23746	14.61
58	19816605	Viral	GS FLX	CF4	Host-associated	Human	sputum	241965	202.41	26268	10.86
59	19816605	Viral	GS FLX	CF5	Host-associated	Human	sputum	194766	221.47	16448	8.45
60	20547834	Viral	GS FLX	Choloroformed	Host-associated	Human	oropharyngeal	214440	203.95	5489	2.56
61	20547834	Viral	GS FLX	Filtered	Host-associated	Human	oropharyngeal	243844	218.64	8297	3.40
62	20368178	Microbial	GS FLX	LF/PP 1dpc group1	Host-associated	Mouse	fecal	34849	224.23	0	0.00
63	20368178	Microbial	GS FLX	LF/PP 7dpc group1	Host-associated	Mouse	fecal	40458	229.36	0	0.00
64	20368178	Microbial	GS FLX	LF/PP 28dpc group1	Host-associated	Mouse	fecal	28795	223.46	4	0.01
65	20368178	Microbial	GS FLX	LF/PP 29dpc group1	Host-associated	Mouse	fecal	34291	223.44	2	0.01
66	20368178	Microbial	GS FLX	LF/PP 35dpc group1	Host-associated	Mouse	fecal	32989	231.09	0	0.00
67	20368178	Microbial	GS FLX	LF/PP 1dpc group2	Host-associated	Mouse	fecal	40608	227.95	1	0.00
68	20368178	Microbial	GS FLX	LF/PP 7dpc group2	Host-associated	Mouse	fecal	32859	228.95	3	0.01
69	20368178	Microbial	GS FLX	LF/PP 28dpc group2	Host-associated	Mouse	fecal	52078	233.12	2	0.00
70	20368178	Microbial	GS FLX	Western 1dpd (29dpc) group2	Host-associated	Mouse	fecal	33983	224.88	9	0.03
71	20368178	Microbial	GS FLX	Western 7dpd (35dpc) group2	Host-associated	Mouse	fecal	34854	219.75	4	0.01
72	20368178	Microbial	GS FLX	Donor	Host-associated	Human	fecal	34203	229.52	2	0.01
73	20668486	Microbial	GS FLX + ABI3730	Assembled fosmids	Host-associated	Kangaroo	fecal	53275	964.14	8	0.02
74	18033299	Microbial	ABI3730 assembled	Termite gut	Host-associated	Termite	content	54992	998	0	0.00
75	15845853	Microbial	ABI3730 (Sanger)	Whale fall 1	Aquatic	Marine	Whale fall	38404	1013.92	0	0.00
76	15845853	Microbial	ABI3730 (Sanger)	Whale fall 2	Aquatic	Marine	Whale fall	38215	987.94	0	0.00
77	15845853	Microbial	ABI3730 (Sanger)	Whale fall 3	Aquatic	Marine	Whale fall	40411	1013.17	0	0.00
78	15845853	Microbial	ABI3730 (Sanger)	Soil	Terrestrial	Soil	Farm soil	138953	1037.93	0	0.00
79	0	Microbial	ABI3730 (Sanger)	PB sand DNA	Terrestrial	Soil	Beach sand	4981	678.58	5	0.10
80	19788654	Microbial	GS FLX	HOT186_25m	Aquatic	Marine	seawater	607109	218.36	116	0.02
81	19788654	Microbial	GS FLX	HOT186_75m	Aquatic	Marine	seawater	660453	206.06	98	0.01
82	19788654	Microbial	GS FLX	HOT186_110m	Aquatic	Marine	seawater	463788	232.34	390	0.08
83	19788654	Microbial	GS FLX	HOT186_500m	Aquatic	Marine	seawater	982172	231.21	119	0.01
84	17921274	Viral	ABI3730 (Sanger)	Chesapeake Bay metagenome	Aquatic	Marine	Virioplankton	5641	694.92	1	0.02
85	18987310	Microbial	ABI3730 (Sanger)	EM	Aquatic	Marine	worm (epibiont)	128909	1143.03	2	0.00
86	16980956	Microbial	ABI3730 (Sanger)	Symbiont metagenome	Host-associated	Gutless worm	marine worm	303633	985.61	1	0.00
87	17878949	Microbial	ABI3730 (Sanger)	Km3	Aquatic	Marine	deep-sea plankton	9046	796.1	1	0.01
88	18441115	Viral	ABI3730 (Sanger)	Bear Paw	Terrestrial	Hot spring	freshwater	7771	985.11	2	0.03
89	18441115	Viral	ABI3730 (Sanger)	Octopus	Terrestrial	Hot spring	freshwater	21356	1004.33	0	0.00
90	18059494	Microbial	ABI3730 (Sanger)	Octopus spring	Terrestrial	Hot spring	microbial mat	20390	979.56	0	0.00
91	18059494	Microbial	ABI3730 (Sanger)	Mushroom spring	Terrestrial	Hot spring	microbial mat	9585	998.29	0	0.00
92	18725995	Microbial	GS FLX	Mid-Bloom DNA-High CO2	Aquatic	Marine	coastal	203825	227.1	8	0.00
93	18725995	Microbial	GS FLX	Mid-Bloom DNA-Present Day	Aquatic	Marine	coastal	130243	231.22	12	0.01
94	18725995	Microbial	GS FLX	Post-Bloom DNA-High CO2	Aquatic	Marine	coastal	324001	174.13	4	0.00
95	18725995	Microbial	GS FLX	Post-Bloom DNA-Present Day	Aquatic	Marine	coastal	294732	225.11	11	0.00
96	19114525	Microbial	GS FLX	Wastewater metagenome	Aquatic	Wastewater	sludge basin	353497	250.97	8	0.00
97	19801459	Microbial	GS FLX	pyrosequencing-derived data set	Terrestrial	Glacier Ice	freshwater	1051604	223.54	339	0.03
98	19892985	Viral	GS FLX	Spring	Terrestrial	Antarctic lake	freshwater	42206	237.82	0	0.00
99	19892985	Viral	GS FLX	Summer	Terrestrial	Antarctic lake	freshwater	39803	220.6	5	0.01
100	19555373	Viral	GS FLX	Effluent DNA	Terrestrial	Reclaimed water	freshwater	255441	245.44	0	0.00
101	19555373	Viral	GS FLX	Effluent RNA	Terrestrial	Reclaimed water	freshwater	202145	222.15	151	0.07
102	19555373	Viral	GS FLX	Nursery DNA	Terrestrial	Reclaimed water	freshwater	275271	243.47	1	0.00
103	19555373	Viral	GS FLX	Nursery RNA	Terrestrial	Reclaimed water	freshwater	244797	232.67	63	0.03
104	19555373	Viral	GS FLX	Potable DNA	Terrestrial	Potable water	freshwater	194282	222.91	7247	3.73
107	20182523	Microbial	ABI3730 (Sanger)	FW106	Terrestrial	Groundwater	freshwater	5996	896.74	0	0.00
108	17355176	Microbial	ABI3730 (Sanger)	GS00a	Aquatic	Marine	Open ocean	642739	1018.7	40	0.01
109	17355176	Microbial	ABI3730 (Sanger)	GS00b	Aquatic	Marine	Open ocean	317180	1012.13	208	0.07
110	17355176	Microbial	ABI3730 (Sanger)	GS00c	Aquatic	Marine	Open ocean	368835	1007.74	127	0.03

MG_ID	PubMed ID	Type (V/M/E)	Sequencing method	Sample name in publication	Biome	Environment	Sub-classification	# Reads (Filtered)	Mean Length (Filtered)	# Contamination C95-I94	% Contamination
111	17355176	Microbial	ABI3730 (Sanger)	GS00d	Aquatic	Marine	Open ocean	332240	1011.14	137	0.04
112	17355176	Microbial	ABI3730 (Sanger)	GS01a	Aquatic	Marine	Open ocean	139964	1005.09	99	0.07
113	17355176	Microbial	ABI3730 (Sanger)	GS01b	Aquatic	Marine	Open ocean	89952	987.18	4	0.00
114	17355176	Microbial	ABI3730 (Sanger)	GS01c	Aquatic	Marine	Open ocean	92351	1003.66	35	0.04
115	17355176	Microbial	ABI3730 (Sanger)	GS02	Aquatic	Marine	Coastal	121590	1058.98	4	0.00
116	17355176	Microbial	ABI3730 (Sanger)	GS03	Aquatic	Marine	Coastal	61605	1086.07	2	0.00
117	17355176	Microbial	ABI3730 (Sanger)	GS04	Aquatic	Marine	Coastal	52959	1074.83	0	0.00
118	17355176	Microbial	ABI3730 (Sanger)	GS05	Aquatic	Marine	Embayment	61131	1079.37	0	0.00
119	17355176	Microbial	ABI3730 (Sanger)	GS06	Aquatic	Marine	Estuary	59679	1082.72	2	0.00
120	17355176	Microbial	ABI3730 (Sanger)	GS07	Aquatic	Marine	Coastal	50980	1087.31	2	0.00
121	17355176	Microbial	ABI3730 (Sanger)	GS08	Aquatic	Marine	Coastal	129655	1062.25	1120	0.86
122	17355176	Microbial	ABI3730 (Sanger)	GS09	Aquatic	Marine	Coastal	79303	1063.36	1078	1.36
123	17355176	Microbial	ABI3730 (Sanger)	GS10	Aquatic	Marine	Coastal	78304	1052.62	8	0.01
124	17355176	Microbial	ABI3730 (Sanger)	GS11	Aquatic	Marine	Estuary	124435	1070.85	20	0.02
125	17355176	Microbial	ABI3730 (Sanger)	GS12	Aquatic	Marine	Estuary	126162	1078.62	26	0.02
126	17355176	Microbial	ABI3730 (Sanger)	GS13	Aquatic	Marine	Coastal	138033	1079.51	2	0.00
127	17355176	Microbial	ABI3730 (Sanger)	GS14	Aquatic	Marine	Coastal	128885	1085.58	34	0.03
128	17355176	Microbial	ABI3730 (Sanger)	GS15	Aquatic	Marine	Coastal	127362	1083.79	61	0.05
129	17355176	Microbial	ABI3730 (Sanger)	GS16	Aquatic	Marine	Coastal	127122	1081.48	8	0.01
130	17355176	Microbial	ABI3730 (Sanger)	GS17	Aquatic	Marine	Open ocean	257581	1091.93	18	0.01
131	17355176	Microbial	ABI3730 (Sanger)	GS18	Aquatic	Marine	Open ocean	142743	1096.2	275	0.19
132	17355176	Microbial	ABI3730 (Sanger)	GS19	Aquatic	Marine	Coastal	135325	1081.94	12	0.01
133	17355176	Microbial	ABI3730 (Sanger)	GS20	Aquatic	Marine	freshwater	296355	1063.42	58	0.02
134	17355176	Microbial	ABI3730 (Sanger)	GS21	Aquatic	Marine	Coastal	131798	1088.44	33	0.03
135	17355176	Microbial	ABI3730 (Sanger)	GS22	Aquatic	Marine	Open ocean	121662	1077.41	15	0.01
136	17355176	Microbial	ABI3730 (Sanger)	GS23	Aquatic	Marine	Open ocean	133051	1079.49	1	0.00
137	17355176	Microbial	ABI3730 (Sanger)	GS25	Aquatic	Marine	Reef	120671	1075.5	5	0.00
138	17355176	Microbial	ABI3730 (Sanger)	GS26	Aquatic	Marine	Open ocean	102708	1061.74	29	0.03
139	17355176	Microbial	ABI3730 (Sanger)	GS27	Aquatic	Marine	Coastal	222080	1068.65	10	0.00
140	17355176	Microbial	ABI3730 (Sanger)	GS28	Aquatic	Marine	Coastal	189052	1084.4	1	0.00
141	17355176	Microbial	ABI3730 (Sanger)	GS29	Aquatic	Marine	Coastal	131529	1093.47	82	0.06
142	17355176	Microbial	ABI3730 (Sanger)	GS30	Aquatic	Marine	Warm seep	358972	1088.28	10	0.00
143	17355176	Microbial	ABI3730 (Sanger)	GS31	Aquatic	Marine	Coastal	436401	1057.91	106	0.02
144	17355176	Microbial	ABI3730 (Sanger)	GS32	Aquatic	Marine	Mangrove	147797	1031.74	21	0.01
145	17355176	Microbial	ABI3730 (Sanger)	GS33	Aquatic	Marine	Hypersaline	692255	1054.1	25	0.00
146	17355176	Microbial	ABI3730 (Sanger)	GS34	Aquatic	Marine	Coastal	134347	1058.45	2	0.00
147	17355176	Microbial	ABI3730 (Sanger)	GS35	Aquatic	Marine	Coastal	140814	1078.3	2	0.00
148	17355176	Microbial	ABI3730 (Sanger)	GS36	Aquatic	Marine	Coastal	77538	1106.01	0	0.00
149	17355176	Microbial	ABI3730 (Sanger)	GS37	Aquatic	Marine	Open ocean	65670	1045.4	13	0.02
150	17355176	Microbial	ABI3730 (Sanger)	GS47	Aquatic	Marine	Open ocean	66023	1035.1	1	0.00
151	17355176	Microbial	ABI3730 (Sanger)	GS51	Aquatic	Marine	Reef	128982	1089.28	2	0.00
152	20573248	Viral	GS FLX Titanium	LIB019	Host-associated	Mosquito	mosquitoes	35093	210.59	474	1.35
153	20573248	Viral	GS FLX Titanium	LIB020	Host-associated	Mouse	brain tissue	13899	166.71	4070	29.28
154	20573248	Viral	GS FLX Titanium	LIB021	Host-associated	Mouse	brain tissue	41189	165.29	10084	24.48
155	20631792	Viral	GS FLX	F1T1.1	Host-associated	Human	fecal	22910	237.48	0	0.00
156	20631792	Viral	GS FLX	F1T1.3	Host-associated	Human	fecal	43335	239.61	0	0.00
157	20631792	Viral	GS FLX	F1T2.1	Host-associated	Human	fecal	42657	240.2	0	0.00
158	20631792	Viral	GS FLX	F1T2.1(R)	Host-associated	Human	fecal	29917	244.69	0	0.00
159	20631792	Viral	GS FLX	F1T2.2	Host-associated	Human	fecal	30711	235.49	0	0.00
160	20631792	Viral	GS FLX	F1T2.3	Host-associated	Human	fecal	27769	238.53	1	0.00
161	20631792	Viral	GS FLX	F1M.1	Host-associated	Human	fecal	48283	244.48	0	0.00
162	20631792	Viral	GS FLX	F1M.2	Host-associated	Human	fecal	24288	246.21	1	0.00
163	20631792	Viral	GS FLX	F2T1.1	Host-associated	Human	fecal	70413	243.26	0	0.00
164	20631792	Viral	GS FLX	F2T1.1(R)	Host-associated	Human	fecal	19340	244.08	1	0.01

MG_ID	PubMed ID	Type (V/M/E)	Sequencing method	Sample name in publication	Biome	Environment	Sub-classification	# Reads (Filtered)	Mean Length (Filtered)	# Contamination C95-I94	% Contamination
165	20631792	Viral	GS FLX	F2T1.2	Host-associated	Human	fecal	40014	244.61	0	0.00
166	20631792	Viral	GS FLX	F2T1.3	Host-associated	Human	fecal	26397	247.12	0	0.00
167	20631792	Viral	GS FLX	F2T2.1	Host-associated	Human	fecal	40342	238.43	0	0.00
168	20631792	Viral	GS FLX	F2T2.1(R)	Host-associated	Human	fecal	41414	241.5	0	0.00
169	20631792	Viral	GS FLX	F2T2.2	Host-associated	Human	fecal	35959	243.62	0	0.00
170	20631792	Viral	GS FLX	F2M.1	Host-associated	Human	fecal	57719	244.04	4	0.01
171	20631792	Viral	GS FLX	F2M.1(R)	Host-associated	Human	fecal	33724	244.67	5	0.01
172	20631792	Viral	GS FLX	F2M.2	Host-associated	Human	fecal	27393	240.91	0	0.00
173	20631792	Viral	GS FLX	F2M.3	Host-associated	Human	fecal	35282	242.34	0	0.00
174	20631792	Viral	GS FLX	F3T1.1	Host-associated	Human	fecal	18371	242.74	0	0.00
175	20631792	Viral	GS FLX	F3T1.2	Host-associated	Human	fecal	19642	242.29	0	0.00
176	20631792	Viral	GS FLX	F3T1.3	Host-associated	Human	fecal	30120	242	1	0.00
177	20631792	Viral	GS FLX	F3T2.1	Host-associated	Human	fecal	53001	236.57	0	0.00
178	20631792	Viral	GS FLX	F3T2.2	Host-associated	Human	fecal	33499	242.3	0	0.00
179	20631792	Viral	GS FLX	F3T2.3	Host-associated	Human	fecal	28680	240.99	0	0.00
180	20631792	Viral	GS FLX	F3M.1	Host-associated	Human	fecal	24835	240.89	1	0.00
181	20631792	Viral	GS FLX	F3M.2	Host-associated	Human	fecal	37385	242.22	0	0.00
182	20631792	Viral	GS FLX	F4T1.1	Host-associated	Human	fecal	24319	243.19	0	0.00
183	20631792	Viral	GS FLX	F4T1.2	Host-associated	Human	fecal	20410	239.47	1	0.00
184	20631792	Viral	GS FLX	F4T1.3	Host-associated	Human	fecal	14362	238	0	0.00
185	20631792	Viral	GS FLX	F4T2.1	Host-associated	Human	fecal	27695	243.16	0	0.00
186	20631792	Viral	GS FLX	F4T2.3	Host-associated	Human	fecal	29647	238.47	0	0.00
187	20631792	Viral	GS FLX	F4M.1	Host-associated	Human	fecal	24715	238.65	0	0.00
188	20631792	Viral	GS FLX	F4M.2	Host-associated	Human	fecal	23461	239.42	0	0.00
189	20631792	Viral	GS FLX	F4M.3	Host-associated	Human	fecal	29888	244.97	0	0.00
190	20631792	Viral	GS FLX	F4M.3(R)	Host-associated	Human	fecal	15926	245.43	0	0.00
191	20631792	Viral	GS FLX	F5T2.1	Host-associated	Human	fecal	47959	242.78	1	0.00
192	20631792	Viral	GS FLX	F5T2.1(R)	Host-associated	Human	fecal	72279	240.97	1	0.00
193	20668239	Microbial	GS FLX	Meconium	Host-associated	Human	fecal	44832	266.12	11	0.02
194	20668239	Microbial	GS FLX	6	Host-associated	Human	fecal	4657	288.71	0	0.00
195	20668239	Microbial	GS FLX	85	Host-associated	Human	fecal	1897	238.51	10	0.53
196	20668239	Microbial	GS FLX	92	Host-associated	Human	fecal	3392	232.73	85	2.51
197	20668239	Microbial	GS FLX	98	Host-associated	Human	fecal	10265	244.98	10	0.10
198	20668239	Microbial	GS FLX	100	Host-associated	Human	fecal	5922	235.97	34	0.57
199	20668239	Microbial	GS FLX	118	Host-associated	Human	fecal	6081	249.81	0	0.00
200	20668239	Microbial	GS FLX	371	Host-associated	Human	fecal	174758	252.41	17	0.01
201	20668239	Microbial	GS FLX	413	Host-associated	Human	fecal	50711	256.75	1	0.00
202	20668239	Microbial	GS FLX	432	Host-associated	Human	fecal	148539	248.25	68	0.05
203	20668239	Microbial	GS FLX	441	Host-associated	Human	fecal	62021	259.76	6	0.01
204	20668239	Microbial	GS FLX	454	Host-associated	Human	fecal	19894	248.28	1	0.01
